# Mitophagy in kidney and lung epithelial cells: molecular mechanisms, crosstalk, and therapeutic interventions

**DOI:** 10.3389/fphys.2026.1782863

**Published:** 2026-05-12

**Authors:** Xiaoxi Zhou, Jing Sun, Lining Miao, Yixian Zhang

**Affiliations:** Department of Nephropathy, The Second Hospital of Jilin University, Changchun, China

**Keywords:** epithelial cells, innate immunity, kidney-lung crosstalk, mitochondrial quality control, mitophagy, organ crosstalk

## Abstract

Mitophagy is a central component of mitochondrial quality control in both renal tubular and alveolar epithelial cells, where mitochondrial homeostasis is essential for barrier integrity, energy supply, and stress adaptation. Increasing evidence indicates that mitophagy is highly context-dependent across kidney and lung diseases: insufficient mitochondrial clearance is commonly linked to persistent mitochondrial dysfunction, epithelial senescence, and fibrotic remodeling, whereas dysregulated or excessive mitophagy may aggravate epithelial vulnerability under severe inflammatory or infectious stress. In this review, we summarize the molecular regulation of epithelial mitophagy, including PINK1/Parkin-dependent and receptor-mediated pathways, and examine its divergent roles in acute and chronic injury states in the kidney and lung. We further discuss a proposed mitophagy-centered framework for kidney–lung crosstalk. Current evidence is strongest for kidney-to-lung communication, particularly through circulating mitochondrial damage-associated molecular patterns and inflammatory mediators after acute kidney injury, whereas lung-to-kidney links remain supported mainly by organ-level inflammatory, hypoxemic, and hemodynamic mechanisms rather than direct evidence of pulmonary epithelial mitophagy-driven renal injury. Overall, the available literature supports mitophagy as an important mechanistic interface in epithelial injury, but not yet as a fully validated bidirectional epithelial axis. Future therapeutic strategies should therefore aim to restore mitophagy homeostasis in a disease- and stage-specific manner rather than uniformly enhancing or suppressing mitochondrial clearance.

## 1 Introduction

The kidney and the lung are anatomically distinct. However, both of these barrier organs are composed of extensive, highly polarized epithelial monolayers, which can maintain internal homeostasis in the face of fluctuating external environments (Nietmann et al., 2022). Renal tubular epithelial cells (RTECs) and alveolar epithelial cells (AECs) constitute the metabolic core of these organs. To sustain active solute transport along steep concentration gradients, RTECs need to consume large amounts of ATP (Natarajan et al., 2021). In comparison, alveolar epithelial type II cells (AECIIs) sustain the high bioenergetic demand necessary to support pulmonary surfactant synthesis and secretion, which is essential for maintaining alveolar stability (Katzen and Beers, 2020). To meet these high energy requirements, both RTECs and AECs are rich in mitochondria; thus, epithelial cell integrity is tightly linked to mitochondrial quality control (Larson-Casey et al., 2020).

Due to the dependence on oxidative metabolism, both of these organs share the same vulnerability. When experiencing ischemia–reperfusion injury, exposure to nephrotoxic or pneumotoxic agents (such as cisplatin or cigarette smoke, CS), or systemic inflammatory and infectious stress, mitochondrial dysfunction leads to excessive reactive oxygen species (ROS) production, bioenergetic failure, and activation of cell death pathways (Li et al., 2018; Li et al., 2025; Xu and Pang, 2025). Under these circumstances, in place of a constitutive housekeeping process, the selective removal of damaged or dysfunctional mitochondria through mitophagy becomes a critical determinant of epithelial survival (Ma et al., 2020; Wang et al., 2023a).

Mitophagy is regulated by a coordinated network that includes the canonical PINK1/Parkin pathway and stress-responsive regulators, such as hypoxia-inducible factor-1α (HIF-1α) (McWilliams and Muqit, 2017; Yang et al., 2024). Under acute injury, rapid activation of mitophagy generally exerts a protective role. By contrast, its dysregulation may contribute to disease progression in a context-dependent manner: insufficient mitophagy is commonly associated with maladaptive repair and fibrosis, whereas in some severe inflammatory or infectious settings, dysregulated mitophagy signaling or a mismatch between mitochondrial clearance and renewal may exacerbate epithelial injury (Cui et al., 2024; Ma et al., 2024; Cao et al., 2025; Küng et al., 2025).

Clinically, kidney and lung dysfunction frequently occur together rather than in isolation. This kidney-lung interaction is particularly evident in critically ill patients. In the intensive care unit, about 30%-50% of patients with AKI may have pulmonary complications at the same time, including acute lung injury (ALI) or acute respiratory distress syndrome (ARDS). This combination of complications is associated with a significant increase in mortality (Doi et al., 2011; Faubel and Edelstein, 2016; Hepokoski et al., 2021). These clinical observations raise the possibility of shared pathogenic pathways between kidney and lung injury, including mitochondrial dysfunction and dysregulation of epithelial mitophagy. We examine the current evidence through the framework of epithelial mitochondrial quality control. In this review, the mitophagy-centered kidney–lung epithelial connection is presented as an integrative conceptual framework intended to organize emerging but uneven evidence, rather than as a fully mechanistically validated signaling axis. We explore potential mechanisms by which injury signals may propagate across organs, while explicitly highlighting instances where the supporting data remain indirect and where causal, epithelial-specific evidence is still lacking. We summarize the molecular mechanisms regulating mitophagy in renal tubular and AECs, discuss regulatory factors of mitochondrial quality control, and analyze context-dependent roles of mitophagy in epithelial injury and repair. We further explore how mitochondrial dysfunction propagates kidney–lung crosstalk and evaluate emerging therapeutic strategies to restore mitochondrial integrity within this crosstalk framework, including pharmacological regulators and nanotechnology-based therapies.

## 2 Molecular framework and contextual regulation of mitophagy in epithelial cells

Mitophagy is a selective form of autophagy that removes damaged or excess mitochondria. In order to ensure that mitochondria are transported to lysosomes for degradation, mitophagy needs to perceive mitochondrial damage, mark dysfunction, and recruit autophagy-related mechanisms (Yim and Mizushima, 2020; Küng et al., 2025). In epithelial cells with high metabolic needs (such as RTECs and AECs), mitophagy is the core mechanism of mitochondrial quality control. By removing dysfunctional mitochondria (which would otherwise produce excessive ROS and trigger cell death pathways), mitophagy helps maintain the bioenergetic stability, epithelial cell integrity, and limits the progression of tissue damage. Therefore, the activation state and regulation ability of mitophagy pathways have become a key determinant of the fate of epithelial cells under acute stress and chronic metabolic challenges (Palikaras et al., 2018; Wang et al., 2023a).

In epithelial cells, mitophagy occurs primarily through two interrelated pathways: the classic ubiquitin-dependent PINK1/Parkin pathway and the receptor-mediated mitophagy pathway activated under conditions such as hypoxia and metabolic stress (Tang et al., 2018; Sachdeva et al., 2019).

### 2.1 Canonical PINK1/Parkin pathway and its modulators

The PINK1/Parkin pathway is currently the most thoroughly studied mechanism for initiating mitophagy, and it is also the main response pathway of cells to the decline of mitochondrial membrane potential (ΔΨm) (Küng et al., 2025). Under physiological conditions, the serine/threonine kinase PTEN-induced putative kinase 1 (PINK1) is continuously imported into mitochondria and rapidly degraded. After the depolarization of mitochondria, the input of PINK1 is blocked, resulting in its accumulation on the outer membrane (Gan and Callegari, 2022). After stabilization, PINK1 recruits and activates the E3 ubiquitin ligase Parkin, which ubiquitinates multiple mitochondrial outer membrane proteins, thereby marking the damaged mitochondria for engulfment by autophagosomes and subsequent lysosomal degradation (Zhao et al., 2017b; Dai et al., 2019).

In order to implement this pathway efficiently, the mitochondrial network needs to be reshaped in advance. The mitochondrial fission mediated by dynamin-related protein-related protein 1 (DRP1) separates dysfunctional mitochondrial fragments from the complete mitochondrial network and promotes the selective removal of these fragments through PINK1/Parkin-mediated mitophagy (Yang et al., 2008; Krzystek et al., 2021; Wang et al., 2025). This step is essential in epithelial cells, because in these cells, the slender mitochondrial network must be dynamically remodeled to achieve selective removal without affecting the overall energy supply (Ma et al., 2020; Jiao et al., 2021).

Recent studies demonstrate that the classic PINK1/Parkin pathway is widely regulated at multiple levels in epithelial cells:

Transcriptional: Vitamin D receptor (VDR) acts as a direct transcription activator for key mitophagy (Gezen-Ak et al., 2023). VDR binds to the promoter regions of *Pink1* to upregulate their transcription. Therefore, VDR deficiency (common in metabolic diseases such as diabetic nephropathy) reduces PINK1 levels and impairs mitochondrial quality control (Yang et al., 2024). Post-transcriptional: As a competitive endogenous RNA, circular RNA circAASS can bind miR-324-3p. By binding to this microRNA, circAASS blocks the inhibition of *Pink1* mRNA, thus increasing the expression of PINK1 and enhancing mitophagy when the epithelial cells are damaged (Ma et al., 2025). Post-translational: Deacetylase Sirtuin 7 (SIRT7) regulates Parkin activity. When sepsis occurs, SIRT7 is directly inhibited by elevated intracellular Zn²+, resulting in an increase in Parkin acetylation. This modification enhances the activity of Parkin E3 ligase, thereby abnormally enhancing protective mitophagy during early sepsis stress (Guo et al., 2024). Metabolite-mediated: Disease-related metabolites can disrupt the classic mitophagic signaling pathway. In the diabetes model, the up-regulation of ceramide synthase 6 will lead to the accumulation of specific ceramides, which directly bind to PINK1 and physically block Parkin’s recruitment, thus preventing the mitophagy cascade reaction (Wang et al., 2023). Inflammatory: Pro-inflammatory mediators can also disrupt the PINK1/Parkin signaling pathway. During sepsis, macrophage migration inhibitory factor is elevated. It can directly bind to PINK1, blocking PINK1-Parkin interactions. This interference inhibits protective mitophagy and aggravates epithelial damage in an inflammatory environment (Li et al., 2024).

In summary, these regulatory layers show that PINK1/Parkin-mediated mitophagy in epithelial cells is not a simple on-off process, but is finely regulated by metabolism, inflammation and transcription signals.

### 2.2 Hypoxia-inducible and receptor-mediated mitophagy pathways

In parallel with the ubiquitin-dependent mitophagy pathway, epithelial cells also utilize receptor-mediated pathways to directly link stressed mitochondria to the autophagy mechanism (Küng et al., 2025). Hypoxia is a particularly effective factor that induces this mitophagy pattern, and is a common feature of renal and pulmonary pathologies, including ischemia–reperfusion injury and chronic tissue remodeling (Wu and Chen, 2015).

The response of cells to hypoxia is regulated by the hypoxia-inducing factor-1α (HIF-1α), which is a major transcription regulatory factor and also plays a central role in the activation of mitophagy (Li et al., 2019; Wang et al., 2022).

A key target of HIF-1α is BCL2/adenovirus E1B interacting protein 3 (BNIP3). Under hypoxic conditions, stabilized HIF-1α binds to the Bnip3 promoter and drives its transcription. BNIP3 can be located in the outer membrane of the mitochondria and interacts directly with LC3 on the autophagosomes (Hanna et al., 2012). Subsequently, the selective removal process of damaged mitochondria will be started. This HIF-1α/BNIP3 pathway constitutes an important protective mechanism against renal fibrosis and epithelial dysfunction (Li et al., 2023). Hypoxia-induced mitophagy has important disease-specific implications. In contrast-induced AKI, pharmacological inhibition of the NOD-, LRR- and pyrin domain-containing protein 3 (NLRP3) inflammasome stabilizes HIF-1α, leading to enhanced BNIP3-mediated mitophagy, reduced RTECs apoptosis, and preservation of renal function (Lin et al., 2021; Li et al., 2023). In addition to hypoxia, the expression of BNIP3 is also directly regulated by VDR, which makes BNIP3 at the intersection of mitochondrial quality control pathways under metabolic and hypoxic conditions (Yang et al., 2024).

**Figure 1:**
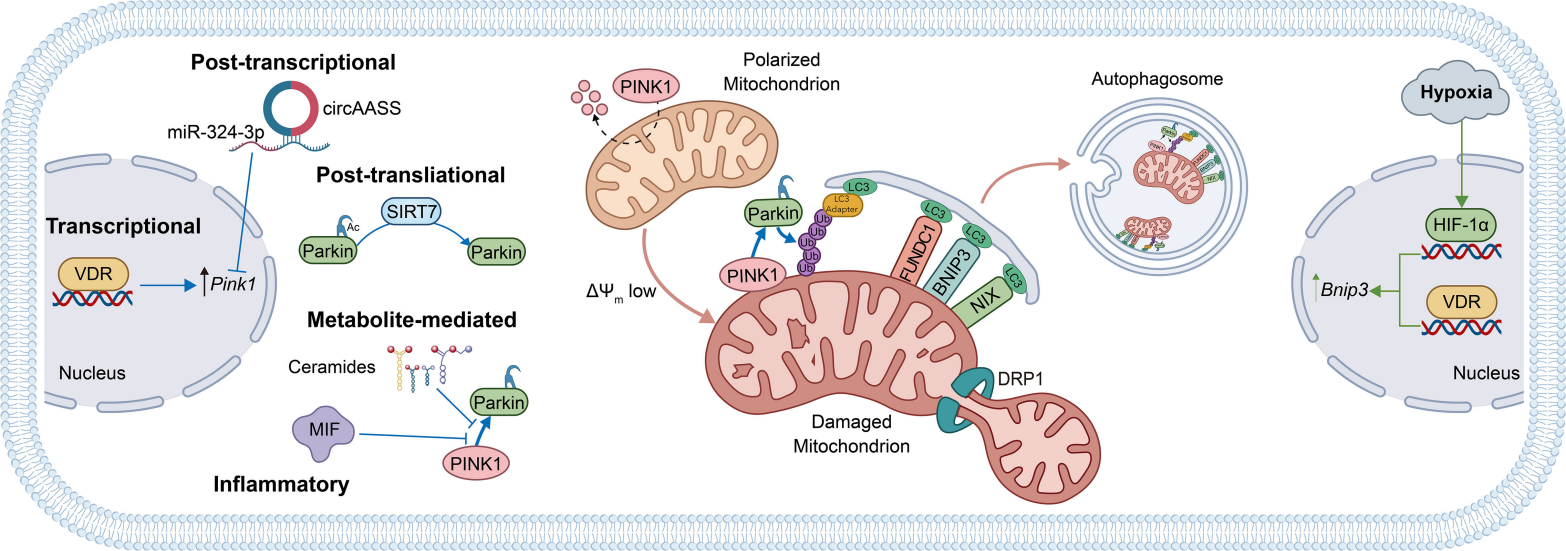
Mitophagy regulatory framework in kidney and lung epithelial cells. Canonical PINK1/Parkin-mediated mitophagy (blue arrows) and receptor-mediated pathways involving BNIP3, BNIP3L/NIX, and FUNDC1 (green arrows) coordinate selective mitochondrial clearance in renal tubular and alveolar epithelial cells. These pathways are modulated by mitochondrial fission and multiple regulatory inputs, including transcriptional, post-transcriptional, and post-translational, etc. PINK1, PTEN-induced putative kinase 1; BNIP3, BCL2 interacting protein 3 like; FUNDC1, FUN14 domain-containing protein 1.

The related mitophagy receptor BCL2 interacting protein 3 like (BNIP3L)/NIX also contributes to mitochondrial clearance in epithelial cells. Accumulation of BNIP3L in RTECs during acute hyperglycemia suggests that mitophagy signaling is initiated under metabolic stress; however, impaired downstream execution of mitophagic flux may limit effective mitochondrial clearance in this context (Wang et al., 2020a). Collectively, receptor-mediated mitophagy pathways complement the canonical PINK1/Parkin system and are particularly relevant under hypoxic and metabolic stress conditions common to kidney and lung disease. Beyond mitophagy initiation, efficient mitochondrial clearance also requires adequate lysosomal capacity. In this regard, transcription factor EB, the master regulator of lysosomal biogenesis and autophagy gene networks, has emerged as an important downstream modulator of mitophagy flux (Park and Lim, 2022; Chen et al., 2024). Activation of transcription factor EB enhances lysosomal function and autophagosome–lysosome fusion, thereby facilitating the degradation of damaged mitochondria once they are labeled by PINK1/Parkin or receptor-mediated pathways (Liu et al., 2021; Fontecha-Barriuso et al., 2022). Collectively, these canonical and receptor-mediated mitophagy pathways, together with their multi-level regulatory inputs, form an integrated mitochondrial quality control network in epithelial cells, as schematically summarized in **Figure 1** for kidney and lung epithelial cells.

For conceptual clarity, in this review we distinguish two broad forms of mitophagy imbalance. Insufficient mitophagy refers to a state in which damaged mitochondria are not efficiently cleared because effective mitophagy flux is inadequate relative to mitochondrial injury burden. By contrast, excessive or dysregulated mitophagy does not simply denote reduced mitochondrial abundance. Rather, it refers to a context-dependent mismatch in which mitochondrial clearance becomes disproportionate to mitochondrial biogenesis and cellular energetic demand, potentially resulting in mitochondrial depletion and epithelial dysfunction. Importantly, reduced mitochondrial mass may also arise from impaired biogenesis, defective mitochondrial renewal, or ongoing epithelial cell death; therefore, static reductions in mitochondrial content should not be interpreted as evidence of excessive mitophagy without appropriate flux-aware and viability-aware assessment (Palikaras et al., 2018; Ma et al., 2020; Yim and Mizushima, 2020; Wang et al., 2023a; Küng et al., 2025).

## 3 Dichotomous roles of mitophagy in kidney diseases

Mitophagy serves as a critical mitochondrial quality control mechanism in the kidney, particularly in RTECs. These cells require abundant ATP to drive reabsorption and concentration (Tang et al., 2021). By removing dysfunctional mitochondria, which excessively produce ROS and activate the cell death pathway, mitophagy maintains the production of ATP, preserves the integrity of the structure of renal tubules, and limits the transmission of injury signals (Yao et al., 2024). Therefore, the activation state and regulation ability of mitophagy pathways have become a key determinant of the fate of renal tubular cells under the conditions of acute injury and chronic metabolic stress.

The role of mitophagy in kidney disease is highly context-dependent, showing significant differences between acute and chronic settings. In AKI, timely mitophagy appears protective in many experimental settings, where it supports epithelial cell survival and tissue repair. However, in the course of the progression of CKD, mitophagy is often insufficient, disordered and maladapted, resulting in failed repair, chronic inflammation and fibrotic remodeling. Next, we will discuss these different functions in detail.

### 3.1 Acute kidney injury: mitophagy as a protective mechanism

In various experimental models, mitophagy activation is a major and important protective response in AKI. By selectively removing damaged mitochondria, mitophagy can reduce the production of mitochondrial reactive oxygen, prevent cell apoptosis and inflammatory cell death, and maintain cell ATP levels, thus promoting epithelial cell survival and tissue repair (Su et al., 2023; Yao et al., 2024).

#### 3.1.1 Ischemia–reperfusion injury

The renoprotective effect of ischemia pretreatment (defined as a short-term, non-fatal ischemia event before a more serious injury occurs) largely depends on the activation of mitophagy (Livingston et al., 2019). Studies show that ischemia pretreatment activates the PINK1/Parkin pathway and receptor-mediated pathways, such as FUNDC1, thus activating the mitochondrial clearance mechanism before prolonged ischemia occurs (Livingston et al., 2019; Wang et al., 2020b).

Early removal of dysfunctional mitochondria can reduce the subsequent ROS burst and limit the propagation of apoptosis signals during reperfusion. In the mouse model of bilateral renal pedicle clamp, mitophagy induced by ischemia pretreatment significantly reduced apoptosis markers, including TUNEL-positive cells and the expression of caspase-3 (Livingston et al., 2019).

#### 3.1.2 Cisplatin nephrotoxicity

Cisplatin-induced nephrotoxicity is characterized by severe mitochondrial DNA (mtDNA) damage and bioenergetic dysfunction in RTECs (Hu et al., 2021). In response to this break, the PINK1/Parkin pathway is activated to eliminate damaged mitochondria (Wang et al., 2018). *In vitro* studies show that silencing Pink1 or Park2 would significantly aggravate cisplatin-induced ATP depletion and more than double the rate of cell apoptosis, underscoring mitophagy as a critical pro-survival mechanism in this context (Zhao et al., 2017b). Effective mitophagy requires DRP1-mediated mitochondrial fission to isolate damaged mitochondrial fragments for selective removal. Thus, inhibition of DRP1 disrupts this process and exacerbates cisplatin-induced injury (Zhao et al., 2017a).

#### 3.1.3 Sepsis-associated AKI

In septic AKI, mitochondrial dysfunction and severe inflammation are the main causes of organ failure. Enhanced mitophagy has been proven to significantly protect the kidney, partly because it inhibits the activation of the NLRP3 inflammasome and limits cell death. Against this background, multiple upstream pathways can promote mitophagy (Lin et al., 2021; Li et al., 2023). It can activate the SIRT1/Parkin pathway and inhibit SIRT7 through the Zn^2+^-dependent signaling pathway, thus jointly enhancing Parkin activity and mitophagy flux (Guo et al., 2024). In the sepsis model induced by cecal ligation puncture, interventions that can trigger autophagy and mitophagy can effectively reverse the pathological elevation of blood urea nitrogen and serum creatinine, which highlights the therapeutic potential of targeted mitochondrial quality control (Guo et al., 2021; Guo et al., 2024).

It is worth noting that cell-based therapies further confirm this principle. In AKI induced by sepsis, systemic infusion of bone marrow–derived mesenchymal stem cells (BMSCs) can upregulate Sirtuin 1 (SIRT1) and Parkin expression in renal tissue, which increases mitophagic flux and protects RTECs from both pyroptosis and apoptosis (Guo et al., 2021). Collectively, these findings suggest that during AKI, adequate mitophagy is crucial for the clearance of ROS-producing mitochondria, the maintenance of PGC–1α–dependent energy metabolism, and the promotion of epithelial cell survival. Nevertheless, in cases of overwhelming injury—such as severe heme protein–induced AKI—even robust mitophagic responses may be insufficient, resulting in collapse of mitochondrial networks and progression of tissue damage (Singh et al., 2022). Although mitophagy is often regarded as a protective mechanism in AKI, its functional effects vary substantially across studies. This heterogeneity likely stems from differences in the injury models employed, such as ischemia–reperfusion injury, cisplatin-induced nephrotoxicity, and sepsis-associated AKI, as well as the varying degrees of metabolic and inflammatory stress placed on renal tubular epithelial cells. Findings from isolated epithelial cell cultures frequently diverge from those in whole-kidney or *in vivo* models, where additional vascular, inflammatory, and systemic factors come into play. Moreover, the timing of assessment along the dynamic injury–repair continuum can further explain apparently conflicting results. Collectively, these observations indicate that the role of mitophagy in AKI is highly context-dependent, rather than uniformly protective across all injury types and stages (Zhao et al., 2017a; Wang et al., 2018; Livingston et al., 2019; Guo et al., 2021; Hu et al., 2021; Singh et al., 2022).

### 3.2 Chronic kidney disease and fibrosis: maladaptation and failed repair

As kidney damage progresses from acute to chronic, the initial protective mechanisms, such as mitophagy, may become impaired or dysregulated. Therefore, maladaptive repair would be prompted. In CKD, insufficient clearance of dysfunctional mitochondria can result in their continuous accumulation. Making these organelles active drivers of disease progression. Chronically damaged mitochondria continuously generate ROS, release damage-associated molecular patterns (DAMPs). These could lead to cellular senescence, sustained inflammation, and tubulointerstitial fibrosis (Bao et al., 2024; Yang et al., 2024).

This failure of mitochondrial quality control is increasingly recognized as a central feature of CKD progression. Experimental and clinical studies show that in models of diabetic nephropathy and obstructive nephropathy, key regulators of mitophagy are frequently downregulated, including *Pink1*, *Park2*, and receptor proteins such as BNIP3L (Zhang et al., 2021). This is correlated with increased oxidative stress and enhanced fibrotic signaling. In this context, pharmacological activation of AMP-activated Protein Kinase (AMPK), supplementation of NAD^+^ precursors, or genetic regulation of mitophagy-related genes to restore mitophagy can alleviate mitochondrial dysfunction, reduce extracellular matrix deposition, and limit fibrotic progression (Fontecha-Barriuso et al., 2022).

The concept of “repair failure” in CKD is closely related to mitophagy dysfunction. When mitochondria are insufficiently renewed, epithelial cells will gradually lose their ability to regenerate and turn into a state of continuous stress, aging or death. The accumulation of dysfunctional mitochondria will continuously activate the mtDNA damage response, promote the transmission of aging-related secretory phenotype signals, and enhance the fibrotic signaling. All of these will lead to the progressive remodeling and functional decline of the renal tubulointerstitium (Cui et al., 2024). Therefore, mitophagy plays a crucial role in the recovery after AKI. However, if there is a chronic deficiency in mitophagy, it becomes a cause of disease. This deficiency can prompt a shift from adaptive repair to irreversible fibrosis and renal failure. The bifold role of mitophagy depends on specific environmental conditions (Lacombe and Scorrano, 2024). It indicates that mitophagy is not only a basal quality-control process, but also plays a key regulatory role in the progression of kidney diseases. Additionally, it is a potential therapeutic target for treating various kidney disorders. Interpretation of mitophagy in CKD is complicated by the marked heterogeneity in disease etiology and stage. For instance, diabetic nephropathy, obstructive nephropathy, and chronic fibrotic kidney disease each impose distinct mitochondrial stresses, potentially eliciting different mitophagy responses. Furthermore, chronic kidney injury involves multiple intertwined processes—including epithelial senescence, persistent inflammation, metabolic reprogramming, and interstitial fibrosis—making it challenging to attribute observed changes solely to epithelial mitophagy defects. Inconsistencies across studies may also arise from differences in experimental models and the specific aspects of mitochondrial quality control examined (e.g., mitophagic clearance versus broader network remodeling). Overall, in CKD, mitophagy appears to function as one component of a broader maladaptive repair program rather than as an isolated driver of disease progression (Zhang et al., 2021; Fontecha-Barriuso et al., 2022; Bao et al., 2024; Cui et al., 2024; Lacombe and Scorrano, 2024; Yang et al., 2024).

## 4 Mitophagy in lung epithelial cells

AECs play a central role in lung homeostasis by forming the extensive surface required for gas exchange and by coordinating epithelial repair after injury (Guillot et al., 2013). Due to their large surface area, continuous exposure to environmental toxins and pathogens, and high metabolic needs related to the production of surfactants and ion transport, AECs are highly dependent on complete mitochondrial quality control mechanisms to maintain cell function and viability (Malsin and Kamp, 2018). Among these mechanisms, the selective removal of damaged or excess mitochondria is a key factor in determining the fate of epithelial cells. Importantly, mitophagy in lung epithelial cells is not just a continuous “sustainable” process. On the contrary, its regulation and functional consequences are highly dependent on the specific environment, from cell protective quality control to direct involvement in epithelial cell damage and cell death, with a wide range of effects (Tsubouchi et al., 2018; Ornatowski et al., 2020).

### 4.1 COPD and emphysema

COPD is characterized by progressive airflow limitation and emphysematous destruction of the lung parenchyma. This is closely associated with mitochondrial dysfunction in AECs (Hoffmann et al., 2019; Tulen et al., 2024). CS is the main pathogenic factor. It has a significant impact on the integrity of mitochondria, and also affects the autophagic signaling pathway of AECs (Vij et al., 2018). Emerging evidence indicates that mitophagy is not always beneficial or harmful to the lung. Its effect depends on its degree, duration and cell environment, and may have completely different or even contradictory effects. Initially, mitophagy may be a protective reaction that can limit mitochondrial damage and maintain the vitality of epithelial cells. However, once exposed to chronic or extreme stress conditions, dysregulated or excessive mitophagy can lead to epithelial cell loss. This usually occurs through programmed cell death pathways, including necroptosis and ferroptosis (Mizumura et al., 2018; Feng et al., 2025). Mitochondrial quality control defects are closely related to the accelerated aging of AECs. This leads to persistent inflammation and tissue degradation unique to emphysema (Tsubouchi et al., 2018).

#### 4.1.1 Cigarette smoke impact on mitophagy flux

Experimental studies show that CS can disrupt the mitophagy process. The degree of disturbance is closely related to specific environmental factors. On the one hand, cigarette smoke extract (CSE) can weaken the protective mitophagy function (Wang et al., 2025). In A549 AECs, exposure to CSE will inhibit the expression of the metabolic regulator SIRT1, which leads to the downregulation of the core mitophagy initiator PINK1. Damage to the SIRT1-PINK1 axis impairs mitochondrial quality control and promotes apoptosis under smoke-induced stress conditions (Guan et al., 2021).

Conversely, multiple studies have shown that long-term or high-intensity CS can induce excessive or pathological mitophagy. The mitochondrial depolarization caused by CS will make PINK1 stabilize on the mitochondrial outer membrane and may activate a form of programmed necrosis that depends on mitophagy. This necrosis mechanism has been shown to be closely related to the development of emphysema (Mizumura et al., 2014; Mizumura et al., 2018). In addition, CSE upregulates the mitophagy receptor FUNDC1 via JNK signaling; excessive activation of FUNDC1 drives ferroptotic cell death in AECs (Feng et al., 2025). Under severe metabolic stress conditions, CS can also activate the adenine nucleotide transporter, resulting in mitochondrial uncoupling and severe ATP depletion (Wu et al., 2020).

In general, these research results show that the overall effect of CS on mitophagy is complex, it depends on several factors. These include the duration and intensity of exposure, cellular metabolic state, and the relative engagement of canonical (PINK1/Parkin) versus receptor-mediated (FUNDC1) pathways. Notably, therapeutic modulation of mitophagy in smoke-related lung injury has yielded promising results. For instance, using urolithin A to help increase mitophagy can reduce oxidative stress and inflammation induced by CS, whereas the inhibition of mitophagy exacerbates epithelial injury. These observations highlight mitophagy as a double-edged yet therapeutically tractable process in CS-related lung disease (Wang et al., 2023b).

#### 4.1.2 Mitophagy deficiency and cellular senescence

Beyond its role in epithelial cell death, insufficient mitophagy appears to be an important contributor to cellular senescence in COPD. Failure to eliminate dysfunctional, ROS-producing mitochondria accelerates the acquisition of a senescent phenotype in AECs (Liu et al., 2025). Reduced expression of the E3 ubiquitin ligase Parkin (*Park2*) has been documented in emphysematous lungs, and Parkin deficiency leads to impaired mitophagy, persistence of damaged mitochondria, and senescence-associated alterations in epithelial cells (Tsubouchi et al., 2018). *In vivo*, Parkin-deficient mice develop exacerbated emphysematous remodeling driven by increased lung epithelial senescence (Ito et al., 2015).

Thus, these findings are not contradictory; rather, each pertains to a different pathological context. In COPD, low levels of mitophagy lead to the accumulation of mitochondria over time, resulting in cell aging and tissue damage. Further, during acute or severe exposure to smoke, excessive mitophagy can lead to the loss of epithelial cells. This process occurs through a regulated path of necrosis (Ahmad et al., 2015). Together, these findings suggest that balanced mitophagy, rather than either extreme activation or suppression, is more consistent with maintaining AEC integrity. By contrast, the apparently divergent roles of mitophagy in COPD likely arise from differences in the intensity and duration of cigarette smoke exposure, the specific epithelial cell types or regional contexts examined, and the experimental models employed. Acute exposure to cigarette smoke extract often unmasks the adaptive stress response of epithelial cells, whereas chronic *in vivo* smoke exposure tends to induce mitochondrial exhaustion, epithelial senescence, and programmed cell death. In addition, studies vary widely in the epithelial compartments assessed—ranging from cultured airway or alveolar epithelial cells to whole-lung tissue with emphysema—which can substantially influence result interpretation. These factors help explain why some studies highlight mitophagy deficiency and mitochondrial accumulation, while others emphasize pathological overactivation during severe injury. Collectively, the role of mitophagy in COPD is best viewed as biphasic and highly model-dependent, rather than uniformly protective or uniformly detrimental (Ahmad et al., 2015; Ito et al., 2015; Tsubouchi et al., 2018; Tulen et al., 2024; Liu et al., 2025).

### 4.2 Idiopathic pulmonary fibrosis

Unlike the bidirectional effects observed in COPD, IPF is a progressive and fatal lung disease characterized by abnormal wound repair, excessive extracellular matrix deposition and irreversible loss of functional alveoli (Podolanczuk et al., 2023). Mounting evidence suggests that mitophagy deficiency or insufficiency is a key contributor to IPF pathogenesis. In this disease, the mitophagy of AECIIs plays a key protective role. When this process is hindered, epithelial cell regeneration may be impaired, and fibrogenesis may be accelerated (Bueno et al., 2020).

#### 4.2.1 AECII dysfunction and impaired regeneration

AECIIs in patients with IPF exhibit pronounced mitophagy defects. This dysfunction is characterized by accumulation of dysfunctional mitochondria and impaired regenerative capacity (Malsin and Kamp, 2018; Wang et al., 2024). A prominent feature of this defect is decreased expression of PINK1, which is a key kinase that activates mitophagy in AECIIs of IPF patients. Both aging-related and chronic endoplasmic reticulum stress - known IPF risk factors – are linked to the PINK1 downregulation. Consistent with these observations, *Pink1* gene-deficient mice spontaneously develop age-dependent pulmonary fibrosis and show increased susceptibility to bleomycin-induced fibrotic injury (Bueno et al., 2015). Persistent mitochondrial dysfunction in Pink1-deficient AECIIs disrupts surfactant metabolism. This kind of dysfunction will aggravate the stress of the endoplasmic reticulum, and also impair the proliferation of epithelial cells and epithelial regeneration after injury (Kim et al., 2020).

#### 4.2.2 Pro-fibrotic signaling and fibrogenesis

Failure of mitophagy in AECIIs has direct consequences for pro-fibrotic signaling. When damaged mitochondria are not efficiently cleared, oxidized mtDNA is released into the cytosol and extracellular space (Bueno et al., 2019). Extracellular mtDNA acts as a potent damage-associated molecular pattern, activating Toll-like receptor 9 (TLR9) and driving NF-κB–dependent induction of transforming growth factor-β, a central mediator of fibrogenesis (Bueno et al., 2019). This mechanistic link was demonstrated by Bueno et al., who showed that mtDNA released from injured AECIIs directly induces Transforming Growth Factor-β production and collagen synthesis (Bueno et al., 2019).

Importantly, restoring mitophagy exerts anti-fibrotic effects in preclinical models. Pharmacological activation of mitophagy with tetrandrine or nanoparticle-mediated delivery of Parkin mRNA significantly reduces collagen deposition and lung fibrosis in bleomycin-injured mice (Chu et al., 2024; Wang et al., 2024). In addition, accumulation of dysfunctional mitochondria due to failed mitophagy promotes a senescence-associated secretory phenotype in AECs, further amplifying pro-fibrotic signaling (Malsin and Kamp, 2018; Chu et al., 2024). Together, these findings support a pathogenic role for defective mitophagy in AECIIs in fibrotic remodeling in IPF. Although a substantial body of evidence implicates defective mitophagy in the pathogenesis of IPF, the relative contribution of mitophagy impairment to fibrotic remodeling remains heterogeneous across models and levels of evidence. Human IPF lung samples show strong correlations with reduced PINK1 expression, mitochondrial accumulation, and epithelial dysfunction; however, these findings cannot reliably distinguish primary, cell-autonomous mitophagy defects in epithelial cells from secondary changes driven by aging, endoplasmic reticulum stress, inflammation, or established fibrosis. Similarly, although experimental models of pulmonary fibrosis yield valuable mechanistic insights, they do not fully recapitulate all histopathological and molecular features of human IPF. Studies also differ in their focus on key parameters—such as PINK1 expression, mitochondrial accumulation, mtDNA release, impaired epithelial regeneration, or profibrotic signaling—which can further influence result interpretation. Collectively, the available data strongly support a pathogenic role for impaired epithelial mitophagy in IPF, while also indicating that its relative contribution compared with other mitochondrial stress pathways is likely model- and stage-dependent (Bueno et al., 2015; Malsin and Kamp, 2018; Tsubouchi et al., 2018; Bueno et al., 2019; Bueno et al., 2020; Kim et al., 2020; Podolanczuk et al., 2023; Chu et al., 2024; Wang et al., 2024).

### 4.3 Acute lung injury and infection

ALI, including ARDS caused by trauma, sepsis, or severe infection (Verma et al., 2025), represents a scenario where mitophagy’s role is finely balanced. On the one hand, removing damaged mitochondria quickly can reduce DAMPs release and epithelial cell death. On the other hand, excessive mitophagy may deplete mitochondrial stores and cause energy failure. Consequently, the outcome of mitophagy activation in ALI depends on the nature of the insult, timing, and cellular context.

#### 4.3.1 Protective versus pathological roles in ALI models

In ALI caused by lipopolysaccharide (LPS), mitophagy can provide protection under certain conditions. The immune-related GTPase IRGM promotes mitophagy in AECs, which is mediated by the PINK1/Parkin pathway. Therefore, mitochondrial integrity is preserved, and the activation of the cGAS–STING inflammatory pathway is suppressed (Shi et al., 2025). Similarly, BCAP31-mediated activation of mitophagy protects AECIIs from LPS-induced apoptosis and epithelial injury (Zhu et al., 2024). Beyond inflammatory injury, hypoxia-induced ALI also engages protective mitophagy. In a hypobaric hypoxia–induced ALI model, PDZ-binding kinase (PBK) activation alleviates lung injury through the SIRT1–PINK1 axis, enhancing mitophagy and preserving mitochondrial function (Sun et al., 2024).

However, if mitophagy becomes overactivated, it can be harmful. In MLE-12 cells, LPS-induced accumulation of mitochondrial citrate binds FUNDC1 and hyperactivates receptor-mediated mitophagy, culminating in necroptotic cell death (Yang et al., 2022). Interventions that restrain excessive mitophagy—such as melatonin treatment or deletion of HDAC3—restore mitochondrial homeostasis and ameliorate lung injury (Ning et al., 2022; Li et al., 2023).

#### 4.3.2 Mitophagy in infection and inflammation

The impact of mitophagy also varies by pathogen. In bacterial pneumonia, strong mitophagy in AECIIs may support epithelial survival and facilitate faster alveolar repair (Suliman et al., 2017). However, once infected by the virus, provoking cytokine storm, such as high Tumor Necrosis Factor alpha levels, can overstimulate mitophagy, potentially depleting mitochondria and exacerbating lung injury. In viral infection–induced ALI models, α7-nicotinic acetylcholine receptor–dependent signaling drives pathological mitophagy (Yu et al., 2024). By regulating mitochondrial integrity and mtDNA release, mitophagy serves as a key player in innate immune signaling pathways, including the cGAS–STING and NLRP3 inflammasome (Sakai et al., 2021; Shi et al., 2025). Therefore, the dysregulation of this process - whether insufficient or excessive - may aggravate lung damage in various cases of inflammation and infection. By contrast, the variable conclusions regarding mitophagy in ALI largely reflect the marked heterogeneity of insults encompassed under this broad term. Lipopolysaccharide exposure, bacterial pneumonia, viral infections, and hypoxic injury impose distinct inflammatory and metabolic stresses on alveolar epithelial mitochondria, and thus do not uniformly elicit the same mitophagy phenotype. Timing of assessment is equally critical: mitophagy can be adaptive during the early phase of mitochondrial stress, whereas in severe or prolonged injury, dysregulated or persistent mitophagy may exacerbate epithelial damage. Furthermore, findings from isolated epithelial cell cultures frequently diverge from those obtained in whole-lung models, which incorporate broader vascular, inflammatory, and immune responses. Collectively, these considerations explain why mitophagy is reported as protective in some ALI contexts yet pathogenic in others, highlighting the importance of interpreting its role according to the specific type of injury, disease stage, and experimental system (Suliman et al., 2017; Ning et al., 2022; Yang et al., 2022; Li et al., 2023; Sun et al., 2024; Yu et al., 2024; Zhu et al., 2024; Shi et al., 2025; Verma et al., 2025).

To facilitate comparison across organs, **Table 1** summarizes the key similarities and differences in mitophagy regulation, functional outcomes, and pathological consequences between renal tubular and AECs.

**Table 1:** Comparison of mitophagy in kidney versus lung epithelial cells.

Aspect	Kidney epithelial cells (RTECs)	Lung epithelial cells (AECs)
Mitochondrial density & demand	Extremely high mitochondrial density and oxygen consumption, supporting solute reabsorption; renders RTECs highly dependent on efficient mitochondrial quality control.	High mitochondrial demand to support surfactant production, barrier maintenance, and post-injury proliferation; necessitates robust mitophagy for epithelial resilience.
Common mitophagy triggers	Ischemia–reperfusion injury, nephrotoxins (e.g., cisplatin), sepsis (systemic inflammation), and metabolic stress (hyperglycemia, hypoxia in kidney medulla).	Inhaled oxidants/toxins (CS, pollution), infections (bacterial pneumonia, viral ARDS), systemic hypoxia (high altitude or ALI), and mechanical stretch (ventilation).
Protective roles of mitophagy	Strongly protective in AKI: removes ROS-producing mitochondria, limits epithelial cell death, supports tubular recovery, and confines injury in reversible insults.	Protective in bacterial infection and selected ALI contexts: preserves barrier integrity, limits inflammasome activation, and supports adaptation to daily oxidative stress.
Pathogenic roles of mitophagy	Predominantly insufficient mitophagy in CKD, leading to mitochondrial accumulation, chronic inflammation, and fibrosis; excessive mitophagy is uncommon but may contribute to epithelial cell death under extreme stress (e.g., prolonged sepsis or ischemia).	Bimodal dysregulation: insufficient mitophagy promotes chronic lung diseases (IPF, COPD) via senescence and fibrogenesis, whereas excessive mitophagy drives acute epithelial injury and cell death during severe infection or sepsis.
Key regulators identified	Enhancers: circAASS (stabilizes *Pink1*), VDR (induces *Pink1*/*Bnip3*), SIRT1 (activates Parkin). Inhibitors: MIF (blocks PINK1–Parkin interaction), specific ceramides (impair Parkin recruitment).	Enhancers: SIRT3 (preserves mitochondrial function), PBK (activates PINK1/Parkin in hypoxic ALI). Stressors: CS (suppresses SIRT1/PINK1 or aberrantly activates FUNDC1-mediated mitophagy), TNF-α (overstimulates mitophagy during viral infection).
Therapeutic modulation	Representative approaches in experimental models include HIF-1α activators (e.g., roxadustat) to induce BNIP3-dependent mitophagy in ischemic injury; AMPK activators (metformin) to restore mitophagy and limit ferroptosis; and mitochondria-targeted antioxidants (MitoTEMPO) to reduce mtDNA damage and improve mitophagy efficiency.	Experimental strategies include antioxidants (e.g., melatonin) to restrain excessive mitophagy in sepsis-associated ALI; PINK1/Parkin enhancers (tetrandrine, ginsenoside Rh2) to restore mitophagy in fibrosis; gene-based delivery (AAV-Parkin or PBK) and anti-inflammatory approaches to interrupt mtDNA-driven cytokine amplification in ARDS.
Predominant evidence level	Predominantly cell and animal studies, with selected human correlative data in AKI and CKD.	Cell and animal studies, complemented by stronger human tissue and clinical correlative evidence in IPF and COPD.
Kidney–lung crosstalk evidence	Kidney-to-lung: relatively direct mtDAMP-based experimental support; the specific contribution of epithelial mitophagy remains incompletely cell-specifically resolved.	Lung-to-kidney: predominantly organ-level inflammatory, hypoxic, and hemodynamic inference; direct pulmonary epithelial mitophagy-specific evidence remains limited.

This table highlights the key similarities and differences in the regulation, functional roles, and pathological consequences of mitophagy in renal tubular epithelial cells and alveolar epithelial cells. It emphasizes the context-dependent balance between insufficient and dysregulated mitochondrial clearance in chronic and acute injury conditions and summarizes the predominant level of evidence supporting each conclusion.

## 5 Mitophagy and kidney–lung crosstalk: a proposed integrative framework

### 5.1 Proposed framework for kidney–lung crosstalk under systemic stress

Clinically, acute or chronic dysfunction in the kidney or the lung can influence the other organ. However, the current evidence does not support a uniformly established epithelial mitophagy-driven bidirectional axis. Rather, available data support a broader framework of kidney–lung crosstalk involving inflammatory mediators, mitochondrial damage-associated molecular patterns (mtDAMPs), hypoxemia, hemodynamic perturbation, and shared epithelial stress responses. At present, the evidentiary basis is asymmetric and should be interpreted accordingly. In the kidney-to-lung direction, direct experimental support is relatively stronger, particularly for circulating mitochondrial danger signals released during kidney injury that can contribute to secondary lung injury. By contrast, in the lung-to-kidney direction, most available evidence remains indirect and is derived mainly from organ-level inflammatory spillover, hypoxic stress, and ventilation-associated hemodynamic mechanisms, with comparatively limited direct evidence linking pulmonary epithelial mitophagy to renal injury. Therefore, the concept discussed here is best viewed as a proposed integrative framework that links epithelial mitochondrial stress to broader inter-organ communication, while direct epithelial-specific causal validation remains limited.

For clarity, the evidence discussed below can be grouped into three levels: (1) direct experimental evidence, in which mitochondrial danger signals or mitophagy-related mechanisms are directly linked to distant-organ injury; (2) supportive organ-level evidence, in which kidney or lung injury is associated with secondary injury in the other organ through inflammatory, metabolic, hypoxic, or hemodynamic pathways; and (3) mechanistic inference, in which epithelial mitophagy is proposed as an upstream integrating process based on convergent but incomplete evidence. This distinction is particularly important when interpreting the relative strength of kidney-to-lung versus lung-to-kidney signaling.

#### 5.1.1 Crosstalk from kidney injury to lung

AKI not only damages the kidney, but also serves as a compelling initiator of systemic inflammation, affecting the interaction between organs. The lung is one of the most commonly affected secondary organs (Lee et al., 2018). Studies show that primary kidney injury can promote ALI, including pulmonary edema, alveolar-capillary barrier destruction and inflammatory cell infiltration (Komaru et al., 2024). In particular, renal ischemia-reperfusion injury has been proven to cause structural and functional damage to the lungs even in the absence of direct lung damage (Hepokoski et al., 2021). One of the main mechanisms connecting the kidneys and lungs involves the accumulation of uremic solutes and toxins, which can usually be removed by the kidneys (Komaru et al., 2024). During AKI, the level of these metabolites increases and circulates in the body, which has a direct toxic effect on pulmonary epithelial cells. For example, protein-bound uremic toxin p-cresyl sulfate accumulates during renal dysfunction (Veldeman et al., 2019). It has been shown that p-cresyl sulfate induces oxidative stress in pulmonary tissue and disrupts the integrity of the alveolar epithelial barrier (Chang et al., 2018). By elevating ROS and inflicting mitochondrial damage, p-cresyl sulfate essentially primes the lung to secondary injury.

In addition to toxins, AKI can also cause a broader systemic inflammatory reaction, thus exacerbating lung damage (Faubel and Edelstein, 2016). Kidney injury leads to the release of DAMPs and pro-inflammatory mediators into the circulation, activating the endothelial cells and epithelial cells of the lungs (Tian et al., 2025). These signals promote neutrophil recruitment, increase vascular permeability, and aggravate alveolar damage, thus linking renal dysfunction to lung inflammation (Hepokoski et al., 2021).

These considerations indicate that AKI should be viewed as a state of systemic inflammatory and metabolic stress, rather than a simple localized kidney event. It causes the accumulation of uremic toxins and releases inflammatory mediators into the systemic circulation. This creates a favorable environment for lung injury and provides a mechanistic explanation for the frequent occurrence of pulmonary complications after AKI. Thus, current support for kidney-to-lung signaling includes direct experimental evidence implicating kidney-derived mtDAMPs in secondary lung injury, whereas the specific contribution of epithelial mitophagy remains mechanistically plausible but incompletely resolved.

#### 5.1.2 Crosstalk from lung injury to kidney

The communication between the kidney and lung is clinically bidirectional, although the mechanistic evidence is not equally developed in both directions. Primary lung injury is also a trigger and amplifier of AKI. Severe lung damage, such as pneumonia, ARDS and ventilator-induced lung injury (Imai et al., 2003; Alge et al., 2021), often damages kidney function even without direct contact with nephrotoxic substances or hypotension. Clinical observations consistently show that patients with severe lung injury carry a high risk of AKI (Panitchote et al., 2019; Park and Faubel, 2021; Cui et al., 2022), which highlights the interdependence between lung function and kidney function.

Severe lung injury can precipitate AKI through both inflammatory and hemodynamic pathways. A “cytokine spillover” from injured lungs releases abundant pro-inflammatory mediators into the circulation, leading to widespread endothelial activation and microvascular dysfunction (Liu et al., 2014). Lung failure impacts overall hemodynamics and oxygenation: sustained hypoxemia and positive-pressure ventilation can worsen renal ischemia and metabolic disturbances (Yang et al., 2020; Marchiset and Jamme, 2022). These factors compound the inflammatory insult, further predisposing the kidney to acute injury. Therapies that attenuate systemic inflammation and oxidative stress during severe lung injury may have the potential to limit secondary renal dysfunction. This concept aligns with emerging evidence of bidirectional lung–kidney interactions and supports the rationale for integrated strategies aimed at mitigating multi-organ injury in critical illness. Notably, the current literature remains asymmetric: kidney-to-lung mechanisms, particularly those involving circulating mtDAMPs and related inflammatory signaling, are supported by more concentrated evidence, whereas lung-to-kidney links are supported mainly by organ-level inflammatory, hypoxic, and hemodynamic mechanisms, with comparatively limited direct evidence linking pulmonary epithelial mitophagy to renal injury.

By contrast, the lung-to-kidney arm remains supported primarily by organ-level inflammatory, ventilatory, and hypoxic mechanisms, with little direct mechanistic evidence showing that altered pulmonary epithelial mitophagy itself drives renal injury.

### 5.2 Molecular mediators of kidney–lung injury: the mitochondrial Bridge

The conversion of a localized kidney or lung injury into a systemic, multi-organ insult state is caused by circulating molecular signals. Several molecules spread inflammation and stress to various organs. Among these, DAMPs and pro-inflammatory cytokines play a central role in connecting kidney and lung injuries. Notably, accumulating evidence suggests that mitochondria-derived DAMPs may act as important mediators in kidney–lung crosstalk.

When mitochondrial quality control is disrupted during AKI, damaged mitochondria release their contents into the circulation. These mitochondrial DAMPs include cell-free mtDNA and mitochondrial transcription factor A. Both of these have strong immune-stimulating effects. Experimental studies provide direct evidence for their pathogenic role. mtDAMPs isolated from injured kidneys could make healthy animals develop lung dysfunction. It is demonstrated that kidney-derived mitochondrial signals can directly provoke lung injury (Hepokoski et al., 2021).

Clinical and experimental observations further support the systemic impact of mtDAMPs. In sepsis-associated AKI, elevated levels of circulating mtDNA—originating predominantly from renal tissue—correlate with increased systemic inflammatory markers, including interleukin-6, and with worse clinical outcomes (Ludes et al., 2021). Mechanistically, extracellular mtDNA activates pattern-recognition receptors, such as the TLR9 (Tsuji et al., 2016). This amplifies inflammatory signaling and contributes to secondary organ damage. Pharmacological or genetic inhibition of mtDNA–TLR9 signaling attenuates this inflammatory cascade, highlighting a causal link between mitochondrial DAMPs release and distant organ injury (Kumar et al., 2025).

Like DAMPs from mitochondrial damage, other circulating mediators can also exacerbate kidney and lung injury. When organs suffer severe damage, small leucine-rich proteoglycans and pro-inflammatory cytokines like interleukin-6 and tumor necrosis factor alpha are released. These factors activate toll-like receptors and inflammasome pathways, such as NLRP3, in both renal and pulmonary tissues. Thereby sustaining a systemic inflammatory milieu that promotes endothelial dysfunction, epithelial injury, and multi-organ failure (Nastase et al., 2018; Zhang et al., 2025).

These findings suggest that kidney–lung crosstalk is induced not only by hemodynamic or metabolic disorders, but also through shared circulating molecular signals. Mitochondrial DAMPs act as a bridge linking impaired mitochondrial quality control in one organ to inflammatory injury in another. This provides a molecular framework for understanding how local epithelial injury escalates to systemic organ dysfunction, but they do not yet establish epithelial mitophagy imbalance as the sole or direct upstream driver of all forms of bidirectional kidney–lung crosstalk. For clarity, the current evidence supporting this proposed framework is further stratified in **Table 2**.

**Table 2:** Evidence stratification for the proposed mitophagy-centered kidney–lung epithelial crosstalk framework.

Direction/Claim	Type of evidence	Current level of support	Main limitation
Kidney → lung: kidney-derived mtDAMPs contribute to secondary lung injury	Direct experimental/preclinical evidence	Relatively stronger	Upstream epithelial mitophagy causality remains incompletely resolved
Kidney → lung: uremic toxins and systemic inflammation amplify lung injury	Supportive organ-level evidence	Moderate	Not mitophagy-specific
Lung → kidney: severe lung injury promotes AKI via cytokine spillover, hypoxemia, and ventilatory/hemodynamic stress	Supportive organ-level evidence	Moderate to strong	Not epithelial- mitophagy-specific
Lung → kidney: pulmonary epithelial mitophagy directly drives renal injury	Mechanistic inference	Limited	Direct epithelial-specific causal evidence lacking

## 6 Therapeutic implications: targeting mitophagy in kidney and lung diseases

**Figure 2:**
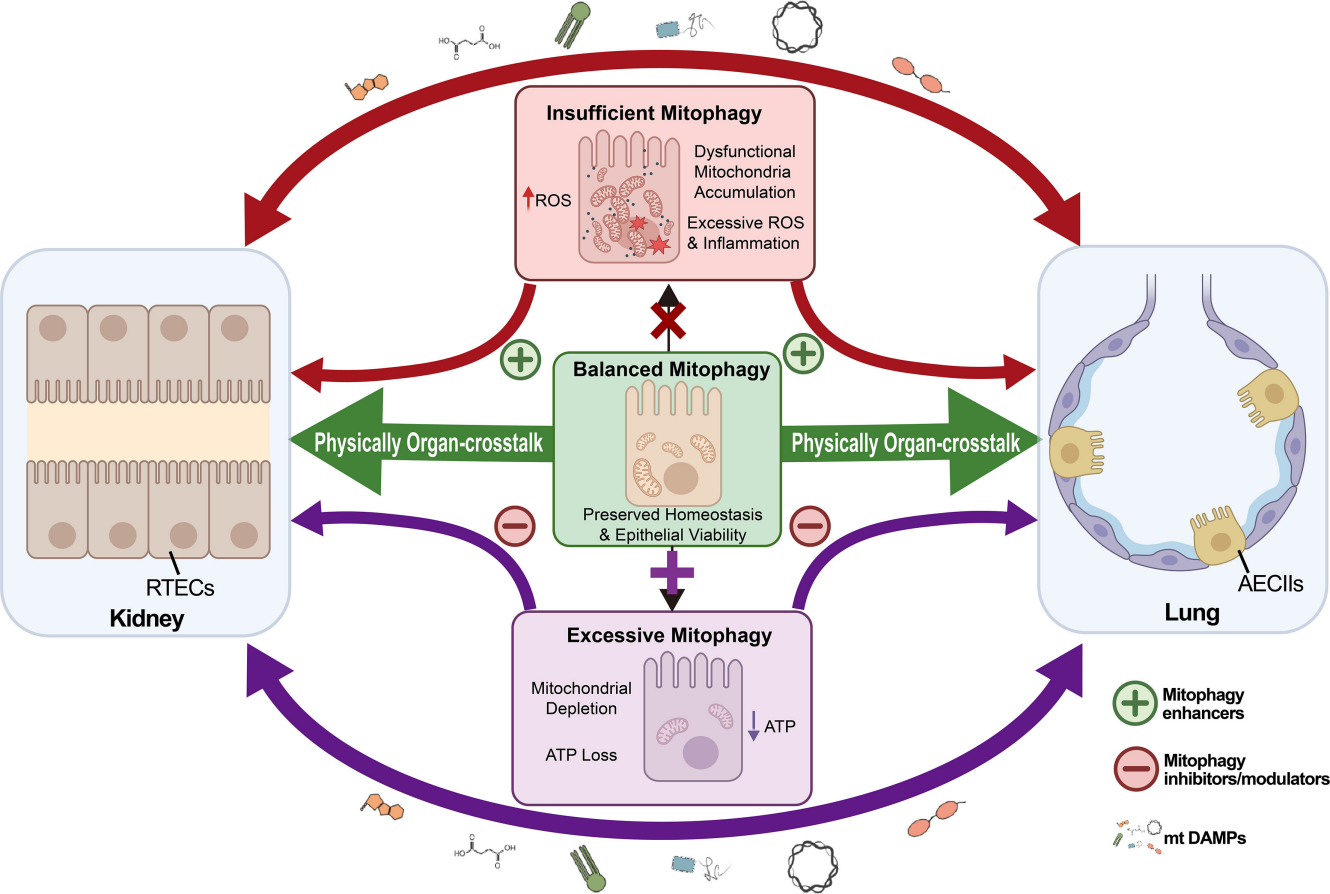
Proposed mitophagy-centered framework for kidney–lung epithelial crosstalk. Balanced mitophagy maintains mitochondrial homeostasis and epithelial integrity in the kidney and lung. Insufficient mitophagy promotes accumulation of damaged mitochondria, oxidative stress, and inflammation, and maladaptive repair, whereas excessive or dysregulated mitophagy may, in selected acute stress settings, lead to mitochondrial depletion and epithelial vulnerability. These processes may contribute to broader kidney–lung crosstalk through circulating inflammatory and mitochondrial damage signals. This model is presented as a hypothesis-generating framework rather than a mechanistically validated bidirectional epithelial axis.

Mitophagy is essential for regulating epithelial cell survival. It also helps manage inflammation and kidney-lung crosstalk. Because of this, controlling mitochondrial quality is a promising target for treating kidney and lung diseases. Crucially, the relationship between mitophagy and disease outcomes is not linear. Insufficient mitophagy can promote mitochondrial dysfunction and tissue injury, while excessive or dysregulated mitophagy can also drive epithelial cell loss (Zong et al., 2024). Therefore, therapeutic strategies must be tailored to restore an appropriate balance of mitochondrial turnover rather than uniformly enhancing or suppressing mitophagy. Building on the context-dependent roles of mitophagy across renal and pulmonary injury states, **Figure 2** illustrates a proposed mitophagy-centered framework for kidney–lung epithelial crosstalk.

### 6.1 Mitophagy-enhancing strategies

In disease settings characterized by impaired mitochondrial quality control, multiple pharmacological interventions have been shown to enhance mitophagy and confer epithelial protection in both renal and pulmonary tissues.

A widely studied pathway is the SIRT1/Parkin pathway, which integrates metabolic sensing with mitophagy activation. In AKI caused by sepsis, systemic administration of BMSCs increases the expression of SIRT1 and Parkin in renal tubular epithelial cells, thereby enhancing mitophagy flux and protecting cells from pyroptosis and apoptosis (Guo et al., 2021). A similar mechanism operates in the lung: sodium tanshinone IIA sulfonate protects AECs from CS–induced apoptosis by upregulating SIRT1 and PINK1, while simultaneously restoring mitochondrial dynamics by increasing fusion and reducing fission (Guan et al., 2021).

Direct activation of the PINK1/Parkin pathway represents another effective approach. In cisplatin-induced AKI, berberine upregulated PINK1 and Parkin, promoted degradation of the autophagy substrate p62, and significantly reduced tubular injury and apoptosis (Qi et al., 2020). In pulmonary fibrosis models, tetrandrine and ginsenoside Rh2 restored deficient PINK1/Parkin-mediated mitophagy in AECs, thereby reducing cellular senescence and fibrotic remodeling (Chu et al., 2024; Zhang et al., 2025). In addition, the myokine irisin, which is downregulated in AKI patients, enhances PINK1/Parkin-mediated mitophagy in ischemia–reperfusion models, suppresses NLRP3 inflammasome activation, and protects renal tubular cells (Cui et al., 2024).

Hypoxia-responsive mitophagy can also be therapeutically leveraged. Roxadustat, a prolyl-hydroxylase inhibitor, stabilizes HIF-1α. It protects against contrast-induced AKI by inducing BNIP3-dependent mitophagy, an effect abolished in *Bnip3*-deficient mice, thereby enhancing receptor-mediated mitochondrial clearance (Lin et al., 2021). Importantly, roxadustat treatment is also effective in hypoxia–reoxygenation–associated renal injury, where HIF-1α activation engages FUNDC1-mediated mitophagy, reduces tubular apoptosis and ROS accumulation, and alleviates ischemic renal damage (Zhang et al., 2024). More broadly, pharmacological activation of the HIF-1α/BNIP3 pathway prior to heatstroke enhanced mitophagy, preserved mitochondrial ultrastructure, and improved survival (Wang et al., 2024a).

Metabolic modulation via AMPK activation further highlights the therapeutic potential of restoring mitophagy. Metformin alleviated diabetic kidney injury by activating AMPK-driven mitophagy and simultaneously inhibiting ferroptosis through suppression of the HIF-1α/myo-inositol oxygenase axis (Wu et al., 2025). Similarly, fibroblast growth factor 5 protects renal tubular epithelial cells from ischemia-like injury by inducing AMPK-dependent mitophagy, reducing ferroptosis and cellular senescence (Lin et al., 2025).

### 6.2 Mitophagy-restraint strategies

In contrast, certain pathological conditions are marked by excessive or maladaptive mitophagy, particularly when mitochondrial clearance outpaces biogenesis and cellular bioenergetic demand. In such contexts, excessive or persistent mitophagy can lead to mitochondrial depletion, bioenergetic failure, and aggravated epithelial injury. However, these findings must be interpreted with caution: reduced mitochondrial content alone does not constitute evidence of excessive mitophagy, as it may also result from impaired biogenesis or ongoing cell death. Within these constraints, therapeutic inhibition of mitophagy has demonstrated protective effects in selected preclinical models (Tian et al., 2022; Yang et al., 2024).

In sepsis-associated ALI, melatonin restrained overactive mitophagy, restored mitochondrial fusion, and preserved metabolic homeostasis in AECs via activation of the SIRT3–SOD2 antioxidant pathway (Ning et al., 2022). Propofol protected AECs from hypoxia-induced autophagic cell death by downregulating HIF-1α and its downstream target BNIP3, thereby preventing excessive mitophagy under sustained hypoxic stress (Ning et al., 2017). In high-altitude pulmonary edema, vitamin D_3_ alleviated lung injury by inhibiting excessive PINK1/Parkin-mediated mitophagy in AECs (Dai et al., 2024). Similarly, in paraquat-induced pulmonary fibrosis, Ginkgo biloba extract reduces pathological mitophagy and epithelial–mesenchymal transition by inhibiting the p38 MAPK signaling pathway (Luo et al., 2025).

These findings together demonstrate that therapeutic mitophagy inhibition aims to preserve essential mitochondria and avert energy collapse. By preventing mitochondrial overclearance, such interventions help maintain cellular ATP levels and function during acute stress.

### 6.3 Mitochondria-targeted antioxidants and redox modulation

Since mitochondrial reactive oxygen species (mtROS) are a key upstream trigger for mitophagy, targeting mitochondrial antioxidants has been explored as an indirect regulator of mitochondrial quality control (Zinovkin et al., 2023). However, experimental evidence shows that mtROS has a dual role: excessive mtROS can cause oxidative damage, and specific ROS signaling is necessary to activate adaptive mitophagy (Fan et al., 2019).

In renal fibrosis models, the mitochondria-targeted antioxidant MitoTEMPO reduced pathological mtROS, restored mitophagy, and attenuated fibrotic phenotypes in renal tubular cells (Li et al., 2023). In contrast, in bleomycin-induced pulmonary fibrosis, scavenging mitochondrial hydroxyl radicals with a targeted antioxidant disrupted PINK1/Parkin-mediated mitophagy, leading to accumulation of damaged mitochondria, enhanced NLRP3 inflammasome activation, and worsened fibrosis (Sakai et al., 2021). These contrasting outcomes show that antioxidant-based therapy must distinguish between pathological ROS and signaling ROS to avoid accidental inhibition of protective mitophagy.

### 6.4 Genetic and advanced therapeutic approaches targeting kidney–lung crosstalk

To achieve greater specificity, emerging therapies adopt gene modulation and targeted delivery systems. Systemic adeno-associated virus serotype (AAV) 9-mediated delivery of the circular RNA circAASS restored mitophagy and mitochondrial biogenesis in AKI models by both enhancing PINK1 expression and stabilizing PGC-1α, resulting in reduced tubular injury and long-term fibrosis (Ma et al., 2025). In pulmonary models, intratracheal AAV delivery of Acetyl-CoA synthetase short chain family member 3 improved metabolic homeostasis in AECs, while AAV5-mediated PBK expression activated SIRT1/PINK1-dependent mitophagy and protected against high-altitude lung injury (Sun et al., 2024; Wang et al., 2024b).

Nanoparticle-based platforms further expand therapeutic possibilities. The “Mito-MEN” system, consisting of Parkin mRNA-loaded nanoparticles tethered to healthy mitochondria, simultaneously replaced dysfunctional organelles and reactivated endogenous mitophagy in fibrotic lungs, markedly reducing collagen deposition and improving lung function (Wang et al., 2024). In addition, systemic BMSC therapy has demonstrated multi-organ protective effects by enhancing mitophagy through secreted factors and extracellular vesicles, benefiting both kidney and lung tissues in systemic inflammatory states (Guo et al., 2021).

Finally, therapies relevant to kidney-lung crosstalk have shown promise. For example, drugs such as green propolis and octreotide reduce kidney and lung damage caused by experimental sepsis by maintaining mitochondrial integrity and inhibiting systemic inflammation (Silveira et al., 2021; Zhang et al., 2025). While these findings primarily document preservation of mitochondrial structure and function, they also raise the possibility that upstream mitochondrial quality control processes may be involved. Experimental evidence further indicates that enhancement of renal mitophagy can limit the release of kidney-derived mitochondrial DAMPs, thereby alleviating secondary lung injury (Hepokoski et al., 2021). Taken together, these observations support the concept that modulation of mitochondrial homeostasis in a primary injured organ may indirectly confer protection to distant organs within the broader kidney–lung crosstalk framework.

## 7 Conclusions and future outlook

This review focuses on mitochondrial quality control, especially mitophagy, as a key factor in determining the integrity of epithelial cells of the kidneys and lungs. Although the physiological functions of renal tubular epithelial cells and AECs are different, they are highly dependent on tightly regulated mitochondrial quality control through pathways such as PINK1/Parkin and HIF-1α/BNIP3. Disruption of these mechanisms can lead to epithelial cell dysfunction in a variety of renal and pulmonary diseases.

A unifying theme derived from this review is that the biological results of mitophagy are highly dependent on the specific environment. Among chronic degenerative diseases, including CKD, IPF and aging-related emphysema, mitophagy deficiency or dysregulation can promote persistent mitochondrial dysfunction, epithelial cell aging, repair failure and progressive fibrosis remodeling. In contrast, under acute explosive stress conditions, such as sepsis or severe smoke damage, dysregulated mitophagy or a mismatch between mitochondrial clearance and renewal may contribute to epithelial vulnerability and cell loss, particularly when bioenergetic reserve is limited. These observations help reconcile the seemingly contradictory roles of mitophagy and emphasize that epithelial fate is determined not by mitophagy per se, but by its timing, magnitude, and coupling to mitochondrial renewal. In addition to organ-specific pathological changes, kidney and lung injuries are also interrelated through systemic mitochondrial signal conduction. The mitochondrial damage-related molecular patterns released by damaged epithelial cells, as well as common hypoxia and inflammatory stress reactions, may contribute to broader kidney–lung crosstalk. These observations suggest that maintaining the mitochondrial integrity of one organ can provide secondary protection for another organ, although direct bidirectional epithelial-specific validation remains limited.

Looking ahead, therapeutic strategies targeting mitophagy must give priority to regulation based on specific situations, rather than simple uniform activation or inhibition. Emerging gene-based approaches, nanotechnology-enabled delivery systems, and precision pharmacological interventions offer promising avenues to restore balanced mitochondrial turnover while minimizing off-target injury. In summary, current evidence suggests mitophagy as an important mechanistic interface between epithelial injury in the kidney and lung and as a promising, though still largely preclinical, therapeutic target whose optimal modulation will depend on disease stage, injury context, and mitochondrial burden.

## Glossary

AKI: Acute Kidney Injury
ALI: Acute Lung Injury
ARDS: Acute Respiratory Distress Syndrome
AECs: Alveolar Epithelial Cells
AECIIs: Alveolar Epithelial Type II Cells
AMPK: AMP-activated Protein Kinase
BNIP3: BCL2/adenovirus E1B interacting protein 3
BNIP3L/NIX: BCL2 interacting protein 3 like
CKD: Chronic Kidney Disease
COPD: Chronic Obstructive Pulmonary Disease
CS: Cigarette Smoke
CSE: Cigarette Smoke Extract
DAMPs: Damage-Associated Molecular Patterns
DRP1: Dynamin-Related Protein 1
FUNDC1: FUN14 domain-containing protein 1
HIF-1α: Hypoxia-Inducible Factor-1α
IPF: Idiopathic Pulmonary Fibrosis
LPS: Lipopolysaccharide
mtDNA: Mitochondrial DNA
mtDAMPs: Mitochondrial Damage-Associated Molecular Patterns
mtROS: Mitochondrial Reactive Oxygen Species
NLRP3: NOD-, LRR- and pyrin domain-containing protein 3
PINK1: PTEN-induced putative kinase 1
RTECs: Renal Tubular Epithelial Cells
ROS: Reactive Oxygen Species
SIRT1: Sirtuin 1
SIRT3: Sirtuin 3
SIRT7: Sirtuin 7
TLR9: Toll-like Receptor 9
VDR: Vitamin D Receptor
